# Exercise Testing in Individuals With Diabetes, Practical Considerations for Exercise Physiologists

**DOI:** 10.3389/fphys.2019.01257

**Published:** 2019-09-27

**Authors:** Christophe Kosinski, Cyril Besson, Francesca Amati

**Affiliations:** ^1^Service of Endocrinology, Diabetes and Metabolism, Lausanne University Hospital and University of Lausanne, Lausanne, Switzerland; ^2^Sports Medicine Center, Lausanne University Hospital and University of Lausanne, Lausanne, Switzerland; ^3^Department of Physiology and Institute of Sport Sciences, University of Lausanne, Lausanne, Switzerland

**Keywords:** cardiopulmonary exercise testing, exercise-induced hypoglycemia, exercise test preparation, insulin, insulin secretagogues, oral anti-diabetic drugs, screening

## Abstract

Exercise and sports activities are crucial for individuals with diabetes. Diabetic patients are often referred to sports clinics for cardiopulmonary exercise testing to evaluate physical capacity, exercise-related symptoms, or to obtain medical clearance. While there is an abundance of literature on cardiopulmonary testing, practical recommendations for exercise physiologists and sports clinic specialists performing exercise testing for this specific population are lacking. The goal of this report is to provide a practical framework to understand, prepare, and perform exercise testing in patients with diabetes, maximizing exercise physiology outcomes, diagnostic value, and ensuring safety.

## Introduction

Cardiopulmonary exercise testing (CPET) is commonly performed in sports medicine (Acsm, 2013; Fletcher et al., 2013). In this particular setting, the goals may differ from CPET performed in cardiology or pneumology clinics (Guazzi et al., 2012). Indications for an exercise capacity evaluation in sports medicine clinics are habitually related to training prescription, to identify causes in performance limitation, or to obtain medical clearance (Riebe et al., 2015). As for the general population, regular physical activity is recommended for individuals with diabetes for overall health (Colberg et al., 2016) as well as to promote better glycemic control (Colberg, 2017). Recommendations vary depending on the type of diabetes and specific individual characteristics such as treatment, habitual training load, and preexisting complications (Colberg et al., 2010, 2016). For each individual, specific and different precautions are necessary before, during, or after physical activity (Younk et al., 2011; Colberg et al., 2016), particularly at high intensity exercise, which is the case during a maximal stress test. This paper is addressed to sports medicine practitioners and exercise physiologists with the goal of helping them prepare and perform optimal exercise testing, maximizing diagnostic value, and ensuring safety.

## Referral

Individuals with diabetes are often referred to sports medicine clinics with the same objectives as non-diabetics: to evaluate physical capacity, for training prescription, to evaluate exercise-related symptoms, and to obtain medical clearance for specific events or high intensity training (Colberg et al., 2016). Diabetologists and diabetes patient educators (American Diabetes Association, 2019a) may also refer their athletic patients to understand glycemic responses to exercise (hypo- or hyperglycemia) or unexplained changes in performance, or simply to increase patient’s education and individual competence, which includes specific dietary and medication adaptations required for different physical activities of various intensities (American Diabetes Association, 2019a). Athletic trainers may want to use CPET to target with precision training heart rate ranges in diabetic athletes given the fact that recent research has shown systematic overestimation of target heart rate in diabetic individuals when applying computation percentages of maximum heart rate or heart rate reserve compared to objective individual markers obtained during a CPET (Moser et al., 2018b). Like with any other exercise testing, the referral purpose should be clear in advance. If the indication for the test is not clear, further information should be obtained by the referring provider.

Guidelines whether or not to perform systematic CPET for diabetic individuals vary according to region and professional associations. In the last recommendations from the American Heart Association (Fletcher et al., 2013), exercise testing is proposed for screening coronary artery disease in asymptomatic individuals who have diabetes based on the fact that diabetes is itself a risk for coronary artery disease. The updated recommendations from the American College of Sports Medicine (Riebe et al., 2015) state that preparticipation health screening should be decided on the basis of the individual’s current physical activity level, the desired exercise intensity, the presence of signs and symptoms, and his medical history (including the presence of diabetes). Both of these recommendations are discussed in the recent position statement from the American Diabetes Association (Colberg et al., 2016), which points to the fact that there is no evidence suggesting that any screening protocol beyond usual diabetes care reduces the risk of exercise-induced adverse events in asymptomatic individuals with diabetes. The authors conclude that pre-exercise medical clearance is not necessary for asymptomatic individuals receiving diabetes care who wish to begin low- or moderate-intensity physical activity not exceeding the demands of brisk walking or everyday living. Individuals who plan to increase their exercise intensity or who meet higher risk criteria, such as their age, diabetes duration, and the presence of additional cardiovascular disease risk factors, may benefit from referral for a medical checkup and possible exercise stress test before starting such activities (Colberg et al., 2010, 2016).

## Contraindications and Screening

The screening procedure and content is the same as for non-diabetic patients (Corrado et al., 2005; Borjesson et al., 2011; Fletcher et al., 2013) with additional specific questions in the history, clinical exam, and context that need to be taken into account in the decision to perform or not a CPET. Absolute and relative contraindications to exercise testing due to cardiovascular conditions (acute myocardial infarction, unstable angina, active endocarditis, etc.) or uncontrolled hypertension are the same for all individuals (Fletcher et al., 2013). Absolute contraindications specific to individuals with diabetes are a poor glycemic control, particularly with high and low excursions that place the patient at risk for a severe hypo- or hyperglycemia, and severe retinopathy that increases the risk of retinal detachment and vitreous hemorrhage during intense exercise (Colberg et al., 2010). All other contraindications are relative.

Nephropathy is not a contraindication *per se*, although in situation of end-renal disease patients in dialyses should be monitored for blood electrolytes (Colberg et al., 2010). As vigorous exercise may lead to false positive readings of microalbuminuria, it is advised not to perform urine check-up on the day after a maximal exercise test. Peripheral neuropathy is not a contraindication to perform exercise testing, but few considerations may need to be taken into account (Colberg et al., 2010). Weight-bearing exercise should be avoided in unhealed ulcers or amputation sites (Colberg et al., 2010). Appropriate footwear for local foot deformity may need adaptations of the exercise test setting. Autonomic neuropathy is a cause of chronotropic incompetence with an attenuated heart rate response to exercise, which will impact termination criteria based on heart rate reserve or theoretical maximal heart rate (Colberg et al., 2010). Autonomic neuropathy may also lead to postural hypotension, alter thermoregulation, and delay gastric emptying, which predisposes to hypoglycemia given unpredictable carbohydrate delivery (American Diabetes Association, 2010, 2019c). If no contraindications are evidenced through history and physical examination, the test has to be prepared carefully in advance to avoid complications.

## Subject Preparation

High intensity exercise in diabetes is metabolically safe if planned correctly (Gawrecki et al., 2017). Planning must be done in the days previous to the CPET. The goal is to be in ideal conditions for the CPET, allowing the patient to perform to maximal capacity, reaching informative exercise and metabolic adaptations, avoiding counter-regulatory mechanisms, and without having to stop the test due to safety risks.

Preparedness includes the avoidance of hypoglycemia, which depends on diabetes medication (Table 1). Exercise-induced hypoglycemia is common in diabetics using insulin or insulin secretagogues (i.e., glinides and sulfonylureas) (Shahar and Hamdy, 2015). Recently, many oral treatments for diabetes have been commercialized with other proprieties than just glycemic control, like kidney or cardiac protection (e.g., SGLT2 inhibitors, GLP1 receptor agonists) (American Diabetes Association, 2019b). Most of these medications have low risk or even no risk of hypoglycemia (Hinnen, 2017; Yang et al., 2017). Identifying the type of medication used is critical to take into account to prepare the CPET.

**TABLE 1:** Risk of exercise-induced hypoglycemia depends on the type of diabetes medication.

**Class and molecules**	**Risk of hypoglycemia**	**CPET precaution and preparation**
**Biguanide**	Low	- No preparation required.
Metformine		- Measures of glycemia before and after CPET for educational purposes.
**DPP4 inhibitors (also called gliptins)**		- Fast-acting oral glucose available.
Sitagliptin, linaglitpin, saxagliptin, vildagliptin, gemigliptin, anagliptin, teneligliptin, alogliptin, trelaglptin, omarigliptin, evogliptin, gosoglptin		
**SGLT2 inhibitors (also called gliflozins)**		
Empagliflozin, dapagliflozin, canagliflozin, ertugliflozin, ipragliflozin, luseogliflozin, tofogliflozin, sotagliflozin^∗^		
**GLP1 receptor agonists (also called incretin mimetics)**		
Exenatide, liraglutide, lixisenatide, dulaglutide, albiglutide, semaglutide		
**Thiazolinediones (also called Glitazones)**		
Pioglitazone, rosiglitazone, lobeglitazone		

**Glinides (also called Meglitinides)**	High	- Preparation required.
Repaglinide, nateglinide, mitiglinide		- Repeated measures of glycemia before, during and after CPET for safety purposes.
**Sulfonylureas**		- Fast-acting oral glucose available.
Chlorpropamide, gliclazide, glimepiride, glibenclamide (= glyburide), glipizide, tolbutamide, tolazamide		
**Insulin and insulin analogs**		
NPH, aspart, lispro, glulisine, detemir, degludec, glargine		

*DPP4, dipeptidyl peptidase 4; SGLT2, sodium–glucose transport protein 2; GLP1, glucagon-like peptide-1; NPH, neutral protamine hagedorn. ^∗^Sotagliflozin is a dual SGLT1/SGLT2 inhibitor. Remarks: A low risk compound in combination with a high-risk medication may further increase the risk. New compounds are tested and approved regularly. Please use caution if a specific molecule is not listed.*

**FIGURE 1:**
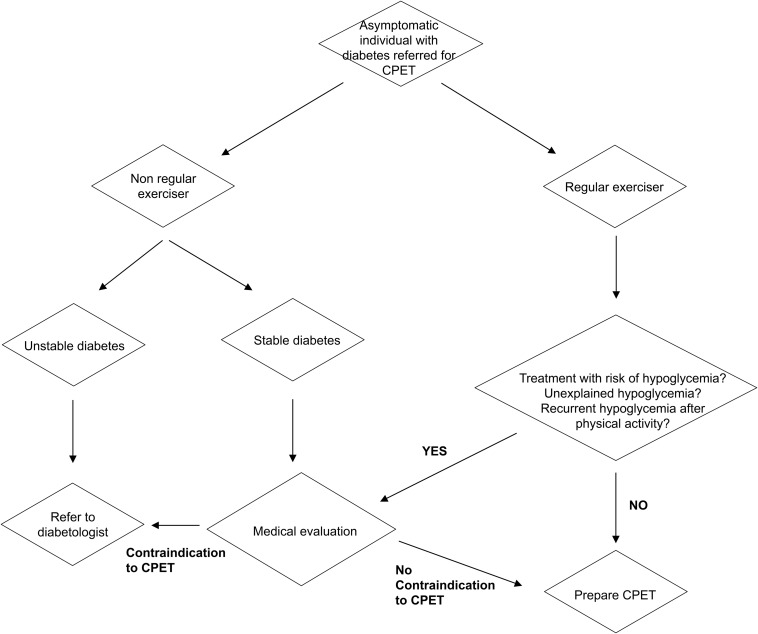
Screening and preparation flowchart for individuals with diabetes referred for a cardiopulmonary maximal exercise test. CPET, cardiopulmonary exercise testing.

In patients treated with insulin, hypoglycemia can happen during, immediately after, or many hours after acute exercise, such as during the following night (Colberg et al., 2016). The risk of hypoglycemia is minimized through multiple strategies including reductions of daily basal insulin dose, reductions of prandial insulin bolus, modification of carbohydrate intake before or during the test (fast acting glucose) or in the meals following the test (complex carbohydrates), inclusion of snacks, and regular glucose checks (Colberg et al., 2016). These strategies are based on individual experiences, such as reducing the long-lasting insulin the night previous to the test, or eating a pre-test meal with specific content and timing, or removing the insulin pump at a specific time before the test. These strategies require advanced planning, preparation, and patient’s know-how. Athletes with diabetes have usually an important knowledge of their own glycemic profile and experience on how to adapt their insulin-therapy or carbohydrate intake to specific exercise durations, intensities, or settings, which is not the case for non-regular exercisers or for patients who have a poor diabetes education or no diabetes care team (Colberg et al., 2016; American Diabetes Association, 2019b). Thus, depending on exercise habits, experience, and diabetes management, we believe that only a subset of diabetic athletes may not require medical screening and preparation guidance before a CPET (Figure 1). As a rule of thumb, any doubt or previous adverse effect of exercise on glycemic control should motivate a medical evaluation to plan and prepare accurately the stress test.

In patients at risk of hypoglycemia (Table 1), avoidance of exercise-induced hypoglycemia is best achieved when glycemia is between 8.3 and 13.9 mmol/L (150–250 mg/dL) at the start of the test (Colberg et al., 2016). Thus, preparation includes anticipating what happens in the last hours or days to get to that specific range at the time of the test. A pre-test blood glucose <5 mmol/L (<90 mg/dL) is a contraindication to the test. The test should be postponed, as even a rapid correction of glycemia will not impair counter-regulatory mechanisms to kick in and impact normal physiological adaptations. A blood glucose range between 5 and 8.3 mmol/L (90–150 mg/dL) is not ideal, the patient should consume additional carbohydrates before starting the test, in amounts depending on the dose and type of insulin that was injected in the hours before the test (between 15 and 30 g of fast acting carbohydrates), thus delaying the start of the test until the desired glycemia is obtained. As an antecedent hypoglycemia severely blunts counter-regulatory responses to exercise, increasing the risk of recurrent hypoglycemia, exercise testing should be avoided for 24 h after an episode of hypoglycemia (Galassetti et al., 2003).

On the hyperglycemia side, a pre-test glycemia between 13.9 and 19.4 mmol/L (250–350 mg/dL) is not a strict contraindication to exercise if ketone bodies are absent (Colberg et al., 2016). If ketosis is detected (>1.5 mmol/L), exercise should be postponed, irrespective of glycemic level, as intense exercise may exaggerate hyperglycemia and ketosis (American Diabetes Association, 2010). Pre-test glycemia >19.4 mmol/L (>350 mg/dL) is a contraindication to intense exercise. Although reports indicate that maximal physical effort can lead to metabolic decompensation, based on incorrect insulin dose (Campbell et al., 2014), increased carbohydrate consumption associated with the fear of hypoglycemia or catecholamine response to intense exercise (Campbell et al., 2014), a recent study has shown that if planned accurately intense exercise is safe in insulin treated diabetics with hyperglycemia >16.6 mmol/L (>300 mg/dL) occurring in 10% of patients while ketone bodies were within reference range (Gawrecki et al., 2017). Nevertheless, how these high glycemia influences physiological outputs and reliance on data acquired during exercise testing is not known and should be taken with caution if the test is used for performance purposes and training prescription. Exercise physiologists and patients should ponder to perform the test in conditions that represent more accurately the athlete’s common situation. At the contrary, if the test is performed to understand glycemic excursions the information acquired with the test may be useful.

Patients should monitor their blood glucose in the days before the CPET and plan to reach the desired glycemic range at the time of the appointment. This may require scheduling the CPET at a moment that is easier to anticipate, such as in the mid-morning or depending on the individual routine. Similarly to non-diabetic individuals, no exercise should be performed before the CPET and the subject should report to the clinic well hydrated with his glucometer, plenty of glucose strips, glucose tablets or his/her preferred fast acting glucose replacement.

To reach the desired glycemia range, two concurrent approaches may be taken into account: therapy adaptation and carbohydrate supplementation (American Diabetes Association, 2002). This is where the patients’ know-how is crucial (Dizon et al., 2019). Insulin therapy is the only treatment that could need an adaptation before CPET and depends on insulin regimen, i.e., multiple daily injections or subcutaneous insulin infusion (pump). Physical activity during CPET is of short duration, but very intense, so basal long acting insulin injections are usually not modified, but again this is unique to each individual and the pharmacodynamics of the different insulins. For rapid acting insulin injections, decreasing dose before or after the test may be needed (Colberg et al., 2016; Riddell et al., 2017). With continuous subcutaneous insulin therapy, basal insulin can be turned off before, during, and/or after the acute bout of exercise. The basal insulin rate may need to be reduced at least 30 min prior to the start of the exercise to allow time for receptor-bound insulin to become inactivated (Kirk, 2009). For other medications with a risk of hypoglycemia (sulfonylureas, glinides), a modification of the dosage is not recommended, but similar to insulin therapy, a particular attention after the test is necessary. If previous exercise induced hypoglycemia has occurred with secretagogues, decreasing dose on exercise days may reduce hypoglycemia risk and thus may be recommended (Colberg et al., 2016; Riddell et al., 2017). Recording meals content, insulin dosage, and glycemia before and after the CPET, even during the night, are usually very informative and allow using the CPET results for educational purposes.

Carbohydrate supplementation is the other option to reach the desired glycemic range before the test (Colberg et al., 2016; Riddell et al., 2017). Guidelines recommend consuming 10 to 15 g of carbohydrate to prevent exercise-induced hypoglycemia, but many studies illustrate that carbohydrate supplementations must be individualized to the type of insulin, i.e., peak insulin levels and duration of insulin action, as well as the absorptive state (Francescato et al., 2004; Dube et al., 2005). Here again, taking the time to discuss test preparation on an individual base is important. Particularly getting to know each individual carbohydrate supplementation habits, early hypoglycemia symptoms and preferred glucose replacement therapy are all important to take into account during the CPET.

## Exercise Test Protocol and Supervision

The test protocol should be selected according to the purpose of testing and the individual patient, similarly to what is done for non-diabetic individuals (Fletcher et al., 2013). Medical supervision follows also the same guidelines as non-diabetic individuals. Exercise testing should be performed under the supervision of qualified health professionals appropriately trained to administer exercise tests and advanced cardiopulmonary resuscitation (Myers et al., 2009). Good communication with the patient is crucial. Test supervisor should know the patient’s specific hypoglycemia symptoms, know how to interpret glycemia modifications during the test, and correct an eventual hypoglycemia. The patient should receive detailed explanations on the purpose of the test, testing procedure, including end points and possible complications if that was not performed during the screening and preparation phase. Supplementary Table 1 provides an example of CPET preparation and supervision form specific to individuals with diabetes.

Measurements of blood glucose levels before, during the exercise test, in the recovery period as well as several hours after the test, are necessary in patients treated with insulin or insulin secretagogues in order to avoid extreme excursions in blood glucose levels (Colberg et al., 2016). Recording of capillary glycemia can be performed alongside with other common measurements such as lactate concentrations, RPE, etc. Capillary glycemia is preferred over the use of subcutaneous continuous glucose monitoring (CGM) for exercise testing as these are still debated for their accuracy during exercise and due to the time lag between the change of blood glucose and its detection by the CGM (Iscoe et al., 2012; Kropff et al., 2015; Moser et al., 2016, 2019). If the patient has a CGM and is using it for his/her usual exercise routine, the combination of CGM and capillary glycemia during the CPET will be useful for education purposes, i.e., to know the tendency and accuracy of the specific CGM device. For clinical settings, real-time glycemia using a well-calibrated strip glucometer is sufficient as the most important information is the relative modification of glycemia. For research exercise labs, the use of a more accurate and precise glucometer using glucose oxidase technology, such as the YSI 2300 (Yellow Springs Instruments Corp., Yellow Springs, OH, United States) coupling the enzymatic assay with oxygen sensors, may be warranted (Amati et al., 2014).

Carbohydrate replacement during and after exercise may help prevent hypoglycemia although it will also impact gas collection and thus impair the proper CPET outcomes. Oral fast acting glucose should be available and ready to be used in the testing room. To treat hypoglycemia (<4 mmol/L or <70 mg/dL) in conscious individuals, 15 g of fast acting glucose should be given orally and repeated if glycemia is still <4 mmol/L after 15–20 min. For regular exercisers, the personnel may want to ask them to bring their habitual fast acting glucose in order to follow what they would do in a real situation.

Like in non-diabetic individuals beta-blockers are known to blunt heart rate response to exercise and lower maximal exercise capacity due to their chronotropic and inotropic negative effects (Sigal et al., 1994). Here, it is also important to know that beta-blockers are thought to induce hypoglycemia unawareness and unresponsiveness, thus a particular attention needs to be brought by the exercise physiologist in patients taking beta blockers (White and Campbell, 2000).

## Interpretation and Training Zones

Interpretation of the CPET outcomes should be done as usual, independently of the presence of diabetes or not (Balady et al., 2010; Guazzi et al., 2012, 2016). Although the debate is still on, studies suggest that highly trained individuals with diabetes can achieve the same maximal cardiopulmonary exercise response as similarly trained subjects without diabetes (Veves et al., 1997; Baldi et al., 2010). These responses are reduced by poor glycemic control despite the fact that training volumes and competition levels are the same as the optimal control group (Baldi et al., 2010). As it is very difficult to match subjects based on exercise physiology outcomes, there are no studies to our knowledge that were able to compare submaximal CPET outcomes in diabetics and non-diabetics while at the same time being able to control for training modalities or habitual physical activity. Most of the studies conclude that poor glycemic control is associated with reduced exercise performance in submaximal intensities (Moser et al., 2017) and modify heart rate dynamics (Moser et al., 2018a). This is of importance for exercise prescription. Submaximal thresholds measured during CPET are valid and objective individual markers for patients with diabetes, while using percentages of maximum values, maximum heart rate, or oxygen uptake, are not sufficient to prescribe exercise intensity individually as almost 63% of their diabetic cohort presented atypical heart rate responses (Moser et al., 2018b). Thus, prescribing by percentages of maximal heart rate or heart rate range not based on individual thresholds may push individuals with diabetes to exercise at too high intensity, inducing different training effects than those desired (Moser et al., 2018b).

## Conclusion

Planned high intensity exercise in well-controlled diabetes is safe, thus CPET too. Diabetes medication is not a contraindication for CPET. A thorough screening is required and includes the search of potential risks for hypoglycemia. The key aspect to obtain valid data is planning to avoid glycemic excursions in subjects with insulin therapy or insulin secretagogues. An important aspect to take into account is that there is no “one size fits all.” Preparing and performing a CPET with an individual with diabetes includes taking into account individualities, know-how, and past experiences. These recommendations are also of interest in other forms of stress tests (e.g., stress electrocardiogram-EKG) without gas exchange analysis. Following-up with the glycemic profile is also required after the test for at least 24 h if the patient presents a risk of hypoglycemia.

## Author Contributions

CK wrote the first draft of the manuscript. CB edited and participated substantially to specific sections. FA edited and wrote the final manuscript. All authors accepted the final version of the manuscript to be published.

## Conflict of Interest

The authors declare that the research was conducted in the absence of any commercial or financial relationships that could be construed as a potential conflict of interest.
